# Doppler Ultrasound for Heart Rate Assessment in a Porcine Model of Neonatal Asphyxia

**DOI:** 10.3389/fped.2020.00018

**Published:** 2020-01-31

**Authors:** Nicolò Morina, Peter A. Johnson, Megan O'Reilly, Tze-Fun Lee, Maryna Yaskina, Po-Yin Cheung, Georg M. Schmölzer

**Affiliations:** ^1^Centre for the Studies of Asphyxia and Resuscitation, Neonatal Research Unit, Royal Alexandra Hospital, Edmonton, AB, Canada; ^2^Department of Pediatrics, Faculty of Medicine and Dentistry, University of Alberta, Edmonton, AB, Canada; ^3^Women and Children's Health Research Institute (WCHRI), University of Alberta, Edmonton, AB, Canada

**Keywords:** infants, newborn, neonatal resuscitation, heart rate, Doppler ultrasound

## Abstract

**Objectives:** Approximately 10% of newborn infants require resuscitation at birth. Accurate heart rate (HR) assessment guides resuscitation interventions, thereby reducing morbidities and mortality. While existing HR assessment methods have several limitations, the Doppler ultrasound (Doppler-US) might be a promising alternative. We aimed to evaluate accuracy and optimal use of Doppler-US for HR assessments during neonatal asphyxia in a pre-clinical model.

**Design:** HR assessments were performed in 16 term newborn piglets that were anesthetized, intubated, and instrumented. Study I evaluated optimal transducer position, Study II compared aortic (AV) and pulmonary (PV) examination modes, and Study III examined accuracy during asphyxia, for HR assessment.

**Setting:** Experimental setting.

**Subjects:** Asphyxia-induced piglets.

**Interventions:** Study I: Doppler-US (USCOM® 1A) HR was assessed on upper (A), middle (B), and lower (C) third of the sternum; study II: Doppler-US HR was assessed using AV and PV examination modes; study III: HR was assessed during asphyxia. Comparisons were made between Doppler-US and the clinical gold standard for HR assessments, electrocardiography (ECG).

**Measurements and Main Results:** Study I: Mean (SD) Doppler-US HR at position A, B, and C showed no difference when compared to ECG HR. Study II: The mean (SD) Doppler-US HR using AV and PV modes also showed no difference when compared to ECG HR. Study III: Bland-Altman analysis revealed a mean difference (95% limits of agreement) between Doppler-US and ECG HR of 1.5 (−16 to 19) bpm. Additionally, motion artifacts produced false peaks and peak size was seen to decrease as bradycardia progressed.

**Conclusions:** HR assessment using Doppler-US during asphyxia is accurate but has limitations and must be further evaluated prior to clinical use. Doppler-US can be positioned along the sternum and use either AV or PV mode for accurate assessments in a piglet model of neonatal asphyxia.

## Introduction

Asphyxia at birth is the most common reason that newborn infants fail to make a successful fetal-to-neonatal transition, as it can depress myocardial function and induce bradycardia, leading to asystole (cardiac arrest) (1). Heart rate (HR) is therefore the most important parameter to assess a newborn infant's clinical status at birth. Assessment of HR is used to determine the timing, type and efficacy of respiratory support and neonatal resuscitation interventions (2, 3). During asphyxia, assessment of HR must be accurate to avoid either overestimation or underestimation, which could either lead to delayed or inappropriate interventions (4, 5). Auscultation, umbilical cord palpation, electrocardiography (ECG), and pulse oximetry (PO) are recommended by the current neonatal resuscitations guidelines for HR assessment at birth (6, 7). However, studies have reported that HR is underestimated by an average of 14 beats per minutes (bpm) during auscultation and 21 bpm during umbilical cord palpation when compared to HR obtained by electrocardiography (ECG) (8, 9). While ECG and PO can measure HR accurately, both are limited by delays in time needed to display first HR values among other issues related to reliability, accuracy, quickness, or ease of use (10, 11).

Doppler ultrasound (Doppler-US) is routinely used for diagnostic and clinical decision making throughout pregnancy and during labor. It uses high frequency sound waves to detect blood flow based on differences in the frequency of emitted and reflected sound waves (12). It is routinely used to assess fetal HR, among other fetal cardiac function parameters, throughout pregnancy and in the delivery room (13, 14). Furthermore, Doppler-US has previously been described to assess HR in the Neonatal Intensive Care Unit and the delivery room (14–17). However, the current evidence for its use for HR assessment during neonatal resuscitation remains scarce. The objectives of this study were to (i) evaluate the accuracy of Doppler-US using different examination modes and transducer positioning and (ii) evaluate its accuracy during progressive bradycardia, using a porcine model of neonatal asphyxia.

## Methods

Sixteen term newborn mixed breed piglets (1–3 days of age, weighing 2.0 ± 0.4 kg) were obtained on the day of experimentation from the University Swine Research Technology Centre. All experiments were conducted in accordance with the guidelines and approval of the Animal Care and Use Committee (Health Sciences), University of Alberta (AUP00002151), presented according to the ARRIVE guidelines (18), and registered at preclincialtrials.eu (PCTE0000161). Animal preparation and maintenance during the experiments is presented as an online supplement.

### Heart Rate Measurements

#### ECG

A 3-lead ECG (Hewlett Packard 78833B monitor, Hewlett Packard, Palo Alto, California, USA) using adhesive leads were placed on the skin at the right forelimb, left forelimb, and left hind limb. ECG was used as gold standard surrogates to compare HR.

#### Ultrasound Cardiac Output Monitor (USCOM) Doppler Ultrasound

The USCOM 1A (Uscom Ltd, Sydney, Australia) utilizes ultrasound waves generated by alternate current in a transducer containing piezoelectric crystals, which creates acoustic energy with a specific frequency in response to vibrations. These waves are then converted into an electronic signal, which is displayed on the monitor along with an audible signal. The USCOM 1A device can detect HR non-invasively by integrating the velocity-time profile of the cardiac ejection flow using either a pulmonary (PV) or aortic (AV) valve examination mode (19–21). It automatically measures flow profile data using the FlowTracer feature, which is used to determine the real-time HR. During the experiment, the Doppler-US audio was disabled to blind the operator from the audible signal.

#### Study Protocol

The studies performed consisted of three objectives: (i) assessment of optimal transducer position, (ii) comparison of AV vs. PV examination mode, and (iii) evaluation of the accuracy of HR assessment during asphyxia.

**Figure 1:**
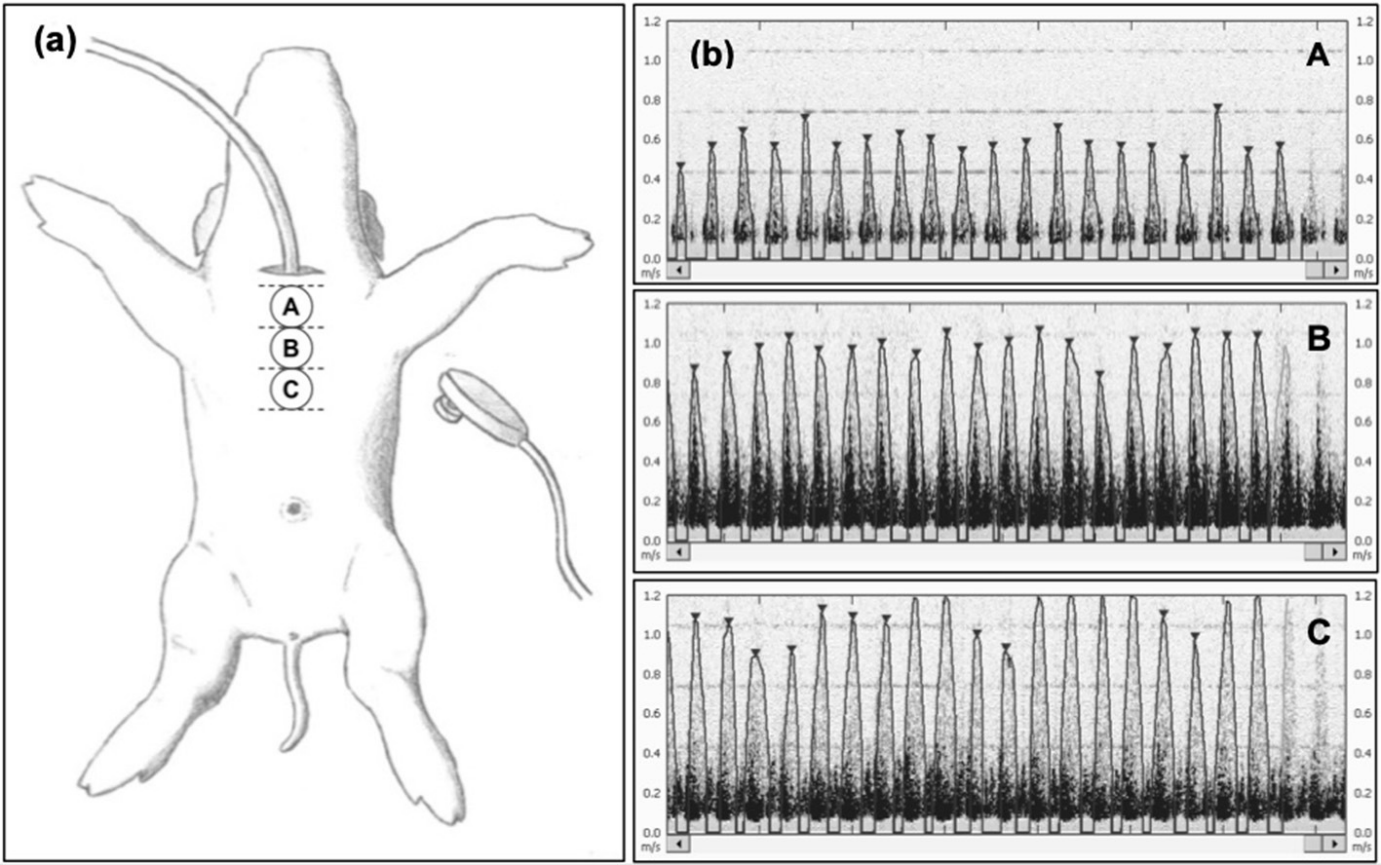
**(a)** Experimental scheme for the assessment of the optimal transducer position to acquire reliable heart rate (HR) data from piglets. The suprasternal notch is not available because of endotracheal intubation. Positions A, B, and C are located at increasing distances from the snout of the animal along the sternal area at 5–6, 6–7, and 7–8 inches (~13–15, ~15–18, and ~18–20 cm, respectively), respectively. For each piglet, a minimum of two HR recordings from each position were carried out by observers blinded from the USCOM monitor during signal acquisition. **(b)** Representative HR outcomes from position A, B, and C, showing no difference in HR detection, but more distinguished and clearer detection of the HR peak signal in position B. The images selected were judged to be best representative of signals obtained during stabilization.

***Study I***: This study was designed to assess optimal transducer position. The sternal area on the chest was divided into three sections: (A) upper (~13–15 cm from the snout), (B) middle (~15–18 cm from the snout), and (C) lower (~18–20 cm from the snout) (Figure 1a). All Doppler-US HR assessments were performed for durations of 10 s per assessment during the stabilization period. A total of 10 participants having no prior experience or formal training with the Doppler-US performed six assessments each (two assessments per transducer position); the sequence of assessments for each participant was randomly determined (e.g., A-C-B-C-B-A).

***Study II***: This study was designed to compare the accuracy of the AV vs. PV examination modes. For this study, only the middle sternum position (position B of study I) was utilized. This position corresponds with the junction of the ascending aorta and aortic arch and the branching of the main pulmonary artery in piglets (22), allowing AV and PV mode assessments. A single operator (GMS) performed 10 assessments with each examination mode (10x AV and 10x PV) in all piglets. All Doppler-US HR assessments were performed for durations of 10 s per assessment during the stabilization period. Multiple assessors were not used in this study due to lack of time during the 1 h of stabilization.

***Study III***: This study was designed to evaluate HR assessment accuracy during asphyxia. After 1 h of stabilization, piglets were subjected to 30 min of hypoxia by decreasing the fraction of inspired oxygen to 10% and decreasing ventilation rate by 10 breaths/min every 10 min. This was followed by asphyxia until asystole, which was achieved by disconnecting the ventilator and clamping the endotracheal tube. Asystole was defined as no audible heart rate during auscultation for at least 6 s and zero carotid blood flow. All HR assessments were performed during the asphyxia time leading to asystole (i.e., between disconnection of the ventilator and clamping of the endotracheal tube until confirmation of asystole). The Doppler-US was operated by a single operator (GMS), who was blinded to ECG HR display, and HR was continuously recorded and assessed every 30 s. Markers were placed within the LabChart program (ADInstruments, Dunedin, New Zealand) to indicate HR assessment times. Post-experiment, the marker was then compared to waveforms from the ECG to determine HR at the time of assessment using Doppler-US. HR as determined by ECG was defined as the gold standard (23). Following confirmation of asystole, HR assessments were ceased and interventions were performed according to the study protocol (24). Multiple operators were not used in this study as piglets were undergoing asphyxia and changing operators was unfeasible.

#### Statistical Analysis

All Doppler-US operators were blinded from the ECG HR and the Doppler-US HR. All statistical analyses were performed by a statistician blinded to the intervention (MY). Data was tested for normality and the level of agreement between the measured Doppler-US HR and the gold standard ECG HR were assessed with Bland-Altman plots (25–27).

***Study I:*** All Doppler-US HR values were obtained by 10 different personnel were recorded and compared to ECG. A one-way ANOVA was conducted to compare transducer positioning. A Bland-Altman plot was also performed for each position to compare ECG and Doppler-US HR values. Moreover, a two-way mixed absolute agreement intraclass correlation coefficient (ICC) was computed as a measure of inter-rater reliability for each position. To adjust for repeated measures taken consequently from the same subject, linear mixed models with random effects were fitted with HR at each position as an outcome and technique (Doppler or ECG) as fixed independent variable. Effect of a subject, time of the measurement, and technique (Doppler or ECG) were also included as random effects in the model. ICC between measurements taken by Doppler and ECG was computed using the formula ρ = (variability between techniques)/(total variability) (28). *Study II:* A one-way ANOVA was conducted to determine differences between AV and PV examination modes for Doppler-US HR assessments, compared to ECG HR. *Study III:* All Doppler-US HR values were recorded by a single assessor. A Bland-Altman plot and one-way ANOVA was conducted to determine to compare ECG and Doppler-US HR values. All *p*-values were two-sided. All statistical analyses were performed using SAS Ver.9.4 (SAS Institute Inc., Cary, NC).

## Results

Baseline parameters before asphyxia are presented in Table 1. The assessments for optimal positioning of Doppler-US transducer and optimal examination mode for HR assessment were performed in 14 piglets, and Doppler-US HR assessment during asphyxia were done in 16 piglets. On average, time required for assessments ranged from 5 to 20 s for all operators.

**Table 1:** Baseline parameters.

***n***	**16**
Sex	
Female	6
Male	10
Weight (kg)	2.08 (1.7–2.4)
Age (days)	1.6 (1–3)
SpO_2_ (%)	97.8 (91–99)
Heart rate (bpm)	171 (143–226)
MAP (mm Hg)	60 (50–79)
CVP (mm Hg)	4 (1–7)
pH	7.51 (7.4–7.6)
PaCO_2_ (torr)	34.7 (27.8–42.9)
PaO_2_ (torr)	101.8 (59–144)
BEcf (mmol/L)	4.4 (−3 to 10)
HCO_3_ (mmol/L)	30.3 (21.2–33.02)

*Continuous data are presented as mean (range)*.

**Figure 2:**
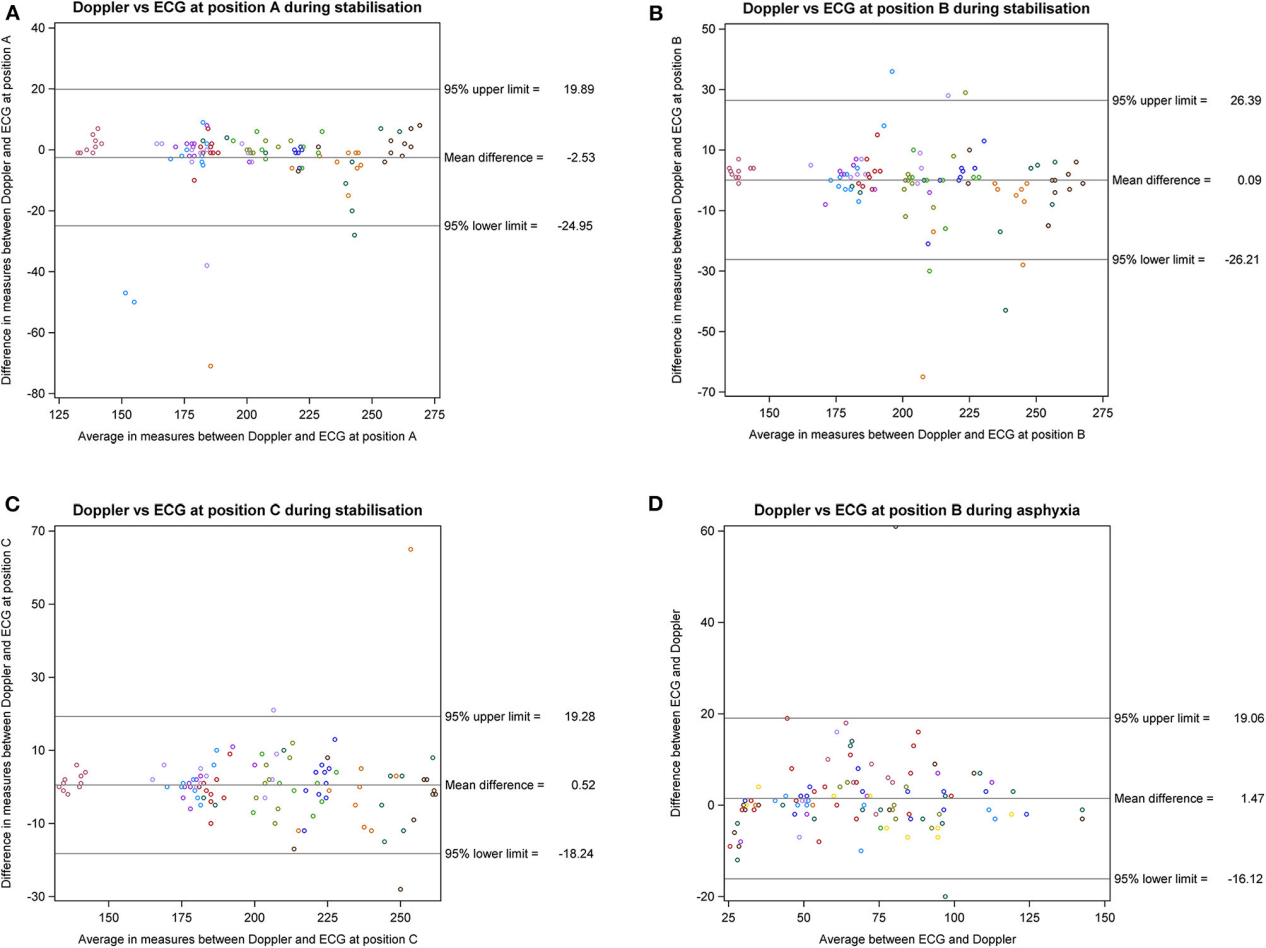
Bland-Altman plots for Studies I and III. **(A–C)** ***Study I:*** Bland-Altman plot with correction for multiple observations per subject for Doppler ultrasound HR from respective positions A, B, or C vs. ECG HR. **(D)** ***Study III:*** Bland-Altman plot with correction for multiple observations per subject for Doppler ultrasound HR vs. ECG HR. ECG, electrocardiography; CBF, carotid blood flow; Doppler, Doppler ultrasound.

*Study I:* A total of 134 assessments were performed with each Doppler-US transducer position during stabilization period after surgical instrumentation. The mean (SD) HR for the Doppler-US at position A, B, and C was 200 (35), 203 (33), and 203 (34) bpm, respectively, compared to ECG HR at position A, B, and C, which was 202 (34), 203 (35), and 203 (34) bpm, respectively (Figure 1). The Bland-Altman plots are visualized in Figures 2A–D. Bland-Altman analysis revealed a mean difference (95% limits of agreement) of −2.53 (−25 to +20), +0.09 (−26 to +26), and 0.52 (−18 to +19) bpm between Doppler-US HR at position A, B, and C, respectively, when compared to ECG HR (Figures 2A–C). When compared to ECG HR, Doppler-US HR was determined to have adjusted ICC values of 0.946, 0.930, and 0.959 for position A, B, and C, respectively.

**Figure 3:**
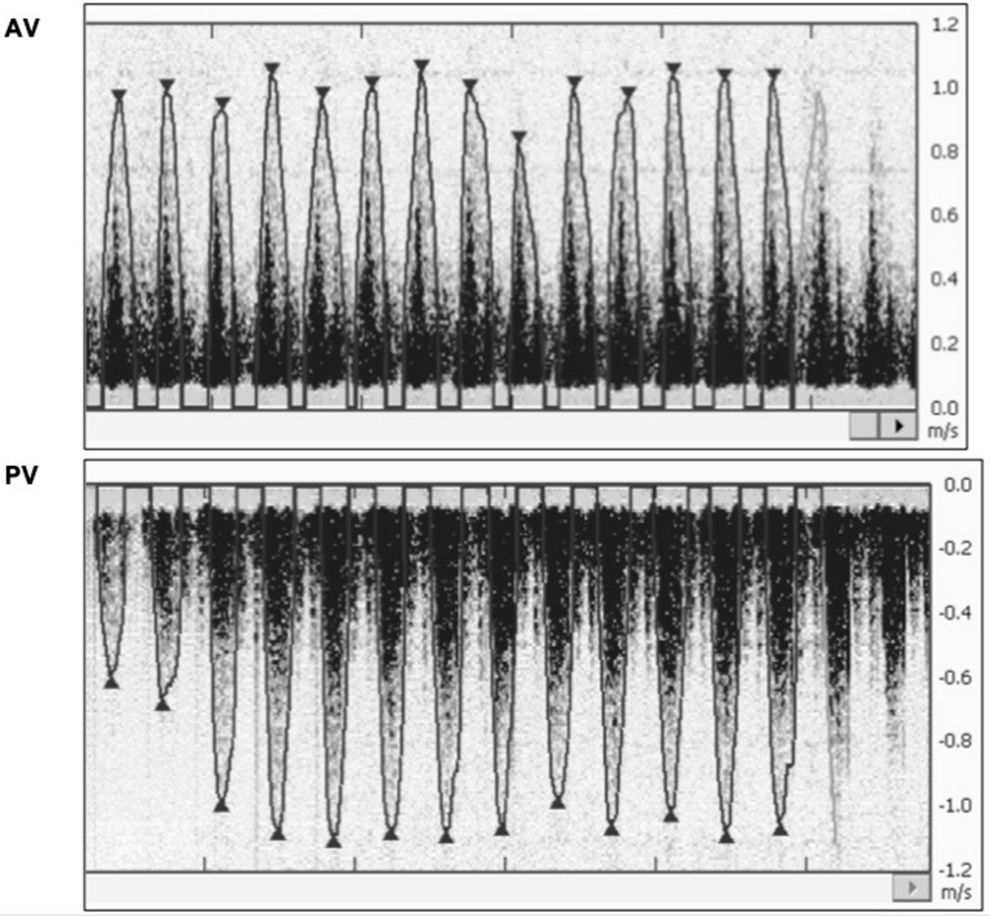
***Study II:*** Representative HR outcomes from aortic valve (AV) and pulmonary valve (PV) examination mode, showing no difference in HR detection, but more distinguished and clearer detection of the HR peak signal in PV mode. The images selected were judged to be best representative of signals obtained during stabilization. ECG, electrocardiography; DUS, Doppler ultrasound; AV, aortic valve examination mode; PV, pulmonary valve examination mode.

*Study II:* A total of 145 assessments were performed with each Doppler-US examination mode during stabilization. The mean (SD) HR for Doppler-US using AV and PV setting was 210 (24) and 211 (20) bpm with ECG HR of 206 (20) and 209 (20) bpm, respectively (AV vs. ECG *p* = 0.29; PV vs. ECG *p* = 0.70) (Figure 3).

*Study III:* The mean (range) time for asphyxia was 369 (72–600) s and a total of 109 assessments, with a median (range) of 8 (2–20) assessments per animal, were made using Doppler-US and ECG. During asphyxia, the mean (SD) HR using Doppler-US was 69 (27) bpm, compared to ECG HR of 70 (28) bpm. The adjusted Bland-Altman analysis revealed a mean difference (95% limits of agreement) of 1.5 (−16 to +19) bpm between Doppler-US and ECG HR (Figure 2D).

**Figure 4:**
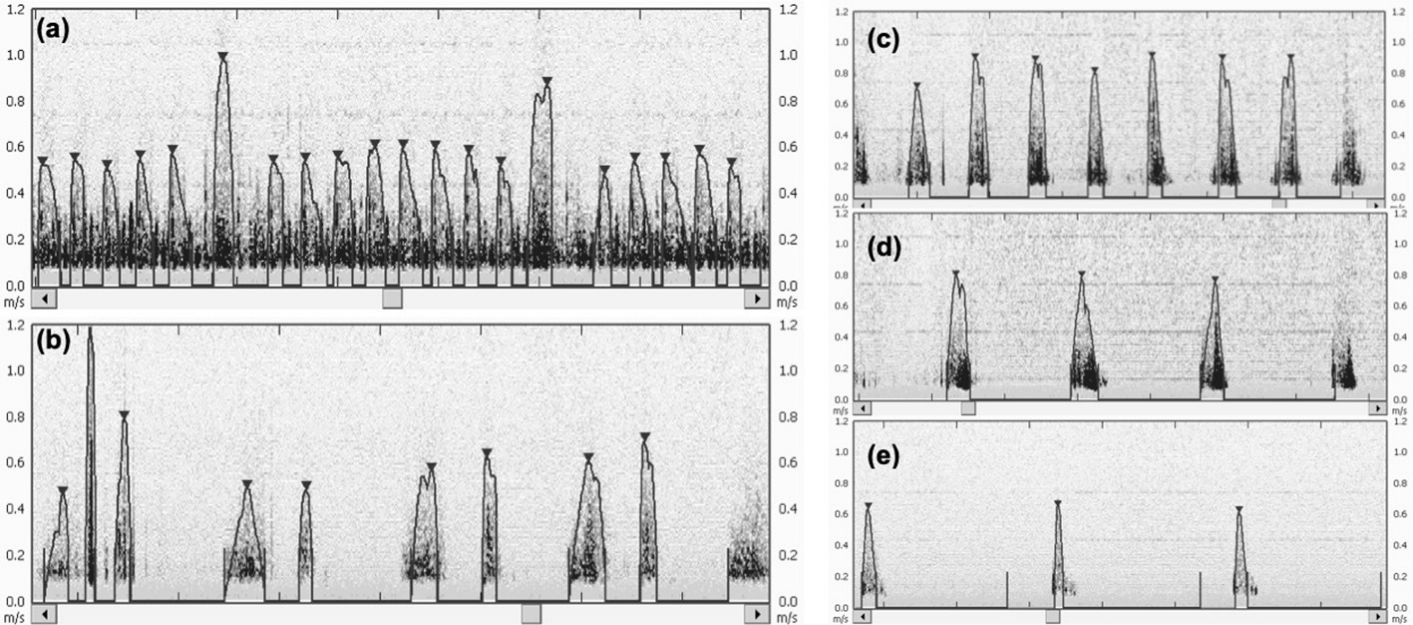
**(a)** Influence of mechanical ventilation on heart rate (HR) signal detection by USCOM. Two distinct higher peaks caused by piglet's emphasized chest movement due to ventilation are observable in the monitor image. The automatic flowtrace function of USCOM calculates the average HR in real-time, but ventilation “peaks” can impair this measurement. In the displayed image, the calculated mean HR is 174 bpm, but after manual removal of the two ventilation-produced signals, this mean HR increases to 182 bpm. **(b)** False positive signals caused by mechanical ventilation and subsequent chest movement in a euthanized piglet. **(c–e)** Heart rate (HR) over the progression of bradycardia during asphyxia with HR at **(c)** 63 bpm, **(d)** 42 bpm, and **(e)** 23 bpm. As shown, the signal appears to lose clarity and decrease in peak size.

In addition, the visualized signals from continuous HR assessment using the Doppler-US were observed to be interfered by mechanical ventilation artifacts (Figure 4a). This was demonstrated by the false positive peaks observed in the euthanized pig (Figure 4b). During asphyxia, peak signal size decreased as bradycardia progressed (Figures 4c–e).

## Discussion

To our knowledge, this is the first study to validate optimal Doppler-US transducer position and examination mode using a porcine model of neonatal asphyxia. Overall, assessing HR from any position along the sternum ~13–20 cm from the snout showed similar accuracy between positions and operator, when compared to ECG HR. Similarly, HR assessment using either AV or PV examination mode also provided similar accuracy when compared to ECG. In summary, Doppler-US for HR assessment during asphyxia is accurate but has limitations, which must be addressed prior to clinical implementation.

There are two positions on the precordium for HR assessment with the Doppler-US in newborn infants: the suprasternal notch (detects blood flow from the aortic valve = AV examination mode), and the left parasternal window (detects blood flow from the pulmonary valve = PV examination mode) (19–21). He et al. previously evaluated AV and PV examination modes in 90 healthy term infants on day one and reported similar accuracy of HR with a mean HR of 125 and 126 bpm, respectively (21). However, HR assessment using the suprasternal notch might be challenging in newborn infants as the position of the Doppler-probe would result in head extension (29), as the Doppler-US probe must be pointed toward the aortic valve. This approach could result in interference during mask ventilation. Our piglet model uses tracheotomy for intubation, which did not allow HR assessment from the suprasternal notch, which is a limitation of the current study. However, our results indicate that HR assessments within the second intercostal space, which corresponds to the junction of the ascending aorta and aortic arch can be used to assess HR with either the PV or and AV mode. This finding has been confirmed by Dyson et al., who reported the best signal for Doppler-US is the second intercostal space over the sternum (16).

Studies examining Doppler-US for HR assessment suggest it is feasible, reliable, accurate, and fast in obtaining HR (14–17), however no previous study has evaluated Doppler-US in asphyxiated infants requiring resuscitation. Although, we observed similar HR with Doppler-US, ECG, or CBF, there was a greater agreement range observed for HR measurements. The increased variability may partially be due to movement of the operator's hand or gasping of the piglet, resulting in loss of signal or interference of the Doppler-US signal. ICC values suggested a low degree of inter-rater variability among operators, suggesting this technique is consistent across different skill levels and clinical expertise. Moreover, the visualization of the FlowTracer feature and heart beat signal provides an additional measure to ensure the reliability of numeric values in a clinical setting. Doppler-US is also much faster compared to ECG when considering the time required for initial assessment as most participants obtained a HR within 5–20 s in our study, compared to the 30–60 s and 60–90 s acquisition for ECG and PO, respectively (30).

A further observation was a Doppler signal during positive pressure ventilation, which was in synchrony with the respiratory rate set on the ventilator (Figures 4a,b). If the clinical team provides positive pressure ventilation during HR assessment using Doppler-US, this interference could be mistaken for a newborn's HR. This might delay critical interventions during resuscitation. Furthermore, with ongoing asphyxia and decreasing cardiac output, a weaker Doppler-US signal was observed (Figures 4c–e), which could also lead to misinterpretation of HR. Doppler-US requires ultrasound gel between the transducer and the skin, therefore any form of movement from the infant, operator or environment could further affect the accuracy of assessed HR. In addition, the ultrasound gel might interfere with chest compression as the chest might become slippery.

Our use of a piglet asphyxia model is a great strength of this translational study, as this model closely simulates the onset of severe asphyxia leading to bradycardia observed during birth asphyxia in the delivery room (31, 32). However, our asphyxia model uses piglets that were sedated/anesthetized (thus with reduced body movements) and limitations such as ventilation artifacts and peak signal detection should be considered before implementing these methods in the delivery room. In addition, assessments during asphyxia were performed by a single operator to reduce bias caused by variations between operators; however, this may limit the generalizability of this study. The use of Doppler-US in the delivery room in asphyxiated infants must also consider the need for a dedicated, skilled personnel for assessments.

## Conclusion

Assessment of heart rate using Doppler ultrasound is accurate during asphyxia. The Doppler ultrasound transducer can provide an accurate heart rate when positioned along the sternum and using either pulmonary or aortic valve examination modes. However, the use of Doppler ultrasound in the delivery room may have limitations including false signals resulting from motion artifacts and ventilation, loss of peak size during severe bradycardia, and greater demand for personnel. Clinical trials are warranted to evaluate the utility of Doppler ultrasound during neonatal asphyxia and resuscitation.

## Data Availability Statement

All datasets generated for this study are included in the article/supplementary material.

## Ethics Statement

All experiments were conducted in accordance with the guidelines and approval of the Animal Care and Use Committee (Health Sciences), University of Alberta (AUP00002151), presented according to the ARRIVE guidelines, and registered at preclincialtrials.eu (PCTE0000161).

## Author Contributions

GS, P-YC, and MO'R: conception. GS, NM, T-FL, MO'R, P-YC, and PJ: data acquisition. GS, NM, T-FL, MO'R, P-YC, PJ, and MY: data analysis, interpreting of results, drafting of the manuscript, critical revision of the manuscript, and final approval of the manuscript.

### Conflict of Interest

The authors declare that the research was conducted in the absence of any commercial or financial relationships that could be construed as a potential conflict of interest.

## Abbreviations

HR: heart rate
Doppler-US: Doppler ultrasound
bpm: beats per minutes
ECG: electrocardiography
PO: pulse oximetry
SpO_2_: oxygen saturation
AV: aortic valve mode
PV: pulmonary valve mode
SD: standard deviation
ICC: intraclass correlation coefficient.
